# Non-invasive chronic kidney disease risk stratification tool derived from retina-based deep learning and clinical factors

**DOI:** 10.1038/s41746-023-00860-5

**Published:** 2023-06-17

**Authors:** Young Su Joo, Tyler Hyungtaek Rim, Hee Byung Koh, Joseph Yi, Hyeonmin Kim, Geunyoung Lee, Young Ah Kim, Shin-Wook Kang, Sung Soo Kim, Jung Tak Park

**Affiliations:** 1 Department of Internal Medicine, College of Medicine, Institute of Kidney Disease Research, Yonsei University, Seoul, Republic of Korea; 2 Division of Nephrology, Department of Internal Medicine, Yongin Severance Hospital, Yonsei University College of Medicine, Yongin, Republic of Korea; 3 Mediwhale Inc, Seoul, Republic of Korea; 4 Singapore Eye Research Institute, Singapore National Eye Centre, Singapore, Singapore; 5 Ophthalmology and Visual Sciences Academic Clinical Program (Eye ACP), Duke-NUS Medical School, Singapore, Singapore; 6 Department of Internal Medicine, International Saint Mary’s Hospital, Catholic Kwandong University, Incheon, Republic of Korea; 7 Albert Einstein College of Medicine, New York, USA; 8 Division of Digital Health, Yonsei University Health System, Seoul, Republic of Korea; 9 Department of Ophthalmology, Institute of Vision Research, Severance Hospital, Yonsei University College of Medicine, Seoul, Republic of Korea

**Keywords:** Risk factors, Prognostic markers

## Abstract

Despite the importance of preventing chronic kidney disease (CKD), predicting high-risk patients who require active intervention is challenging, especially in people with preserved kidney function. In this study, a predictive risk score for CKD (Reti-CKD score) was derived from a deep learning algorithm using retinal photographs. The performance of the Reti-CKD score was verified using two longitudinal cohorts of the UK Biobank and Korean Diabetic Cohort. Validation was done in people with preserved kidney function, excluding individuals with eGFR <90 mL/min/1.73 m^2^ or proteinuria at baseline. In the UK Biobank, 720/30,477 (2.4%) participants had CKD events during the 10.8-year follow-up period. In the Korean Diabetic Cohort, 206/5014 (4.1%) had CKD events during the 6.1-year follow-up period. When the validation cohorts were divided into quartiles of Reti-CKD score, the hazard ratios for CKD development were 3.68 (95% Confidence Interval [CI], 2.88–4.41) in the UK Biobank and 9.36 (5.26–16.67) in the Korean Diabetic Cohort in the highest quartile compared to the lowest. The Reti-CKD score, compared to eGFR based methods, showed a superior concordance index for predicting CKD incidence, with a delta of 0.020 (95% CI, 0.011–0.029) in the UK Biobank and 0.024 (95% CI, 0.002–0.046) in the Korean Diabetic Cohort. In people with preserved kidney function, the Reti-CKD score effectively stratifies future CKD risk with greater performance than conventional eGFR-based methods.

## Introduction

Chronic kidney disease (CKD) is a leading cause of cardiovascular disease and non-communicable disease mortality^1–3^. CKD prevalence is growing rapidly due to an aging global population and increased prevalence of hypertension and diabetes, two major causes of CKD^4,5^. Since CKD is an irreversible condition, prevention is a key factor in decreasing CKD-related morbidity and mortality.

The current approach to CKD screening is based on measuring the estimated glomerular filtration rate (eGFR, calculated from serum creatinine) or examining urine for proteinuria^6^. However, recent evidence indicates that these biomarkers are suboptimal for kidney disease early detection^7,8^. Predicting kidney damage is difficult, especially in people without blood or urine test abnormalities. In addition, risk stratification based on eGFR, which incorporates age and serum creatinine levels, can be misleading in younger, older, pregnant, overweight, or muscular individuals^9^. Similarly, amount of urine proteinuria is also affected by various factors^10^. Moreover, screening adherence tends to be low due to the invasive nature of collecting blood samples^11^.

Retinal photography, a non-invasive and widely utilized diagnostic test, provides information on not only the eye but also the systemic vasculature. The kidney and eye are both highly vascularized organs and share common developmental, physiological, and pathogenic pathways. Damage of one organ often indicates damage to the other which is typically noticeable in hypertensive and diabetic conditions^12–14^. Recently, artificial intelligence application was shown to be capable of providing biomarker estimates, including creatinine, which also led to effective detection of prevalent CKD^15–17^.

In this study, we develop a non-invasive CKD risk stratification tool (“Reti-CKD” score) for people with preserved kidney function, hypothesizing that subtle retinal vasculature changes provide information for future CKD development risk. This is done by applying deep learning algorithms trained on 158,216 retinal photographs and incorporating clinical factors. Internal and external validation in the UK Biobank and Korean Diabetes Cohorts show that the Reti-CKD score effectively stratifies CKD development risk and its predictive performance is superior to traditional eGFR-based methods.

## Results

### Characteristics of the study population

The clinical characteristics of the participants are shown in Table 1 and Supplementary Table 1. In the Korean health screening data (*n* = 79,108) used for the development of the deep-learning algorithm, mean age was 49.5 (standard deviation [SD], 11.8) years and mean eGFR was 100.3 (SD, 14.3) mL/min/1.73 m^2^.

**Table 1:** Baseline characteristics of study participants.

	Health screening data (*n* = 79,108)	UK Biobank (*n* = 30,477)	Korean Diabetic Cohort (*n* = 5014)
Age, mean (SD), years	49.5 ± 11.8	54.2 ± 8.1	55.1 ± 10.5
Male, No. (%)	44,364 (56.1)	13,617 (44.7)	2814 (56.1)
Diabetes, No. (%)	5349 (6.7)	1293 (4.2)	5014 (100)
Hypertension, No. (%)	NA	4050 (13.3)	1400 (27.9)
eGFR, mL/min/1.73 m^2^	100.3 ± 14.3	99.4 ± 6.6	102.5 ± 9.1

*CKD* chronic kidney disease, *eGFR* estimated glomerular filtration rate, *NA* not available, *SD* standard deviation.

In the UK Biobank, among 30,477 participants, 720 (2.4%) were diagnosed with CKD during a mean follow-up duration of 10.8 (interquartile range [IQR], 10.7–11.0) years. Additionally, in the Korean Diabetic Cohort, among 5,014 participants, 206 (4.1%) were diagnosed with CKD during a follow-up duration of 6.1 (IQR, 4.0–8.4) years. The mean eGFR was 99.4 (SD, 6.6) mL/min/1.73 m^2^ in the UK Biobank and 102.5 (SD, 9.1) mL/min/1.73 m^2^ in the Korean Diabetic Cohort. The characteristics of individuals in the UK Biobank and Korean Diabetic Cohort according to Reti-CKD quartiles are provided in Supplementary Tables 2 and 3.

### Saliency maps

**Fig. 1:**
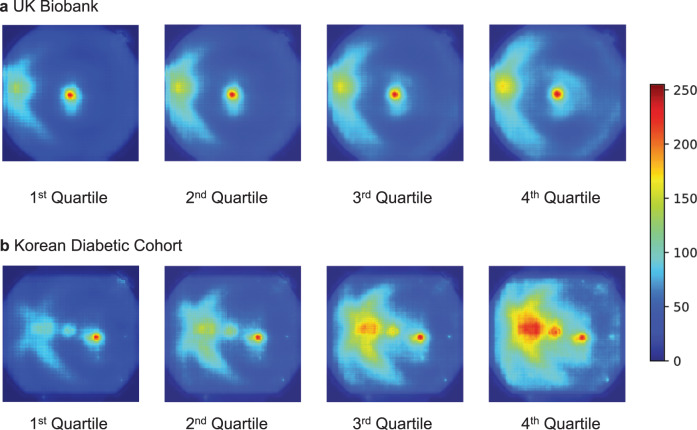
Augmented saliency map according to deep-learning-derived retina-CKD probability. Augmented saliency maps according to quartiles of deep-learning-derived retina-CKD probability in the **a** UK Biobank and **b** Korean Diabetic Cohort are shown. Saliency is represented in color-scale (scaled between 0 and 255). Highlighted areas along the arcade vessels were more prominent in images with higher deep-learning-derived retina-CKD probability.

Aggregated saliency maps indicated that the highlighted areas along the arcade vessels were more prominent in images with higher deep-learning-derived retina-CKD probability (Fig. 1).

### Reti-CKD score performance

**Fig. 2:**
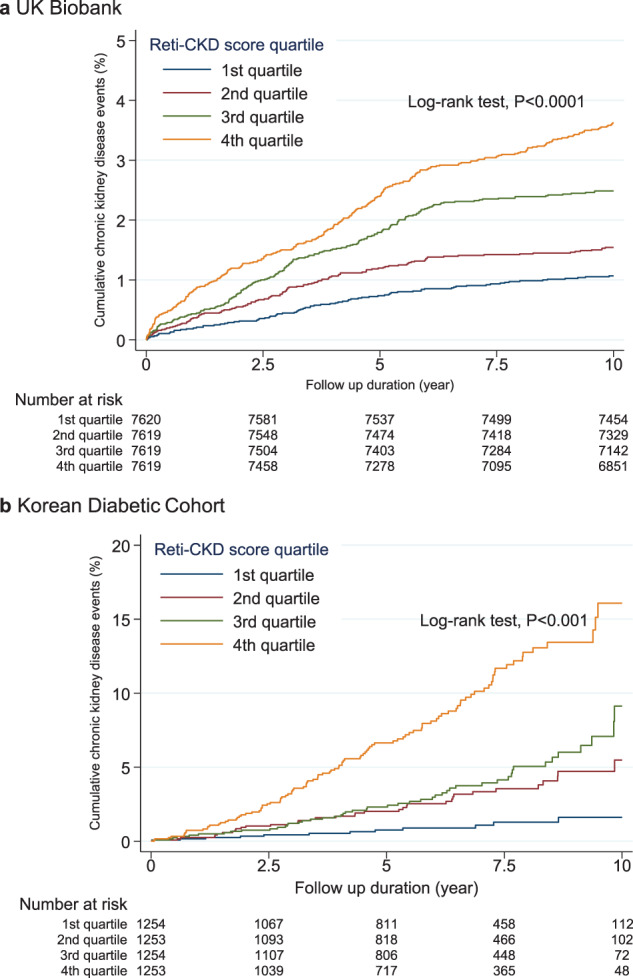
Cumulative incidence of chronic kidney disease events according to Reti-CKD score quartiles in the UK Biobank and Korean Diabetic Cohort. Cumulative chronic kidney disease (CKD) incidences are illustrated according to the Reti-CKD score quartile in the **a** UK Biobank and **b** Korean Diabetic Cohort. There was a clear association between CKD development and the Reti-CKD scores in both cohorts.

Kaplan–Meier curves of the longitudinal validation sets are presented in Fig. 2. During the mean 10.8-year follow-up period, 321,417 person-years were examined in the UK Biobank. In the Korean Diabetic Cohort, during the mean 6.1-year follow-up period, 30,122 person-years were examined. Kaplan–Meier curves showed distinct CKD risk stratifications based on the Reti-CKD score quartiles in both cohorts.

The hazard ratios (HRs) of CKD incidence showed a dose-dependent association across the quartiles. The adjusted HRs per one SD increment of Reti-CKD score were 1.34 (95% confidence interval [CI], 1.27–1.41) in the UK Biobank and 1.94 (95% CI, 1.63–2.31) in the Korean Diabetic Cohort (Table 2).

**Table 2:** Risk of chronic kidney disease according to Reti-CKD scores.

Reti-CKD score	CKD events/N	Person-years	Incidence rate^a^	Crude model HR (95% CI)	*p* value	eGFR-adjusted model^b^ HR (95% CI)	*p* value
UK Biobank							
1st Quartile	83/7620	81,801	1.0 (0.8–1.3)	1 (reference)		1 (reference)	
2nd Quartile	125/7619	81,065	1.5 (1.3–1.8)	1.69 (1.29–2.23)	<0.001	1.49 (1.12–1.97)	<0.001
3rd Quartile	205/7619	80,042	2.6 (2.2–2.9)	2.60 (2.01–3.36)	<0.001	2.45 (1.87–3.20)	<0.001
4th Quartile	307/7619	78,508	3.9 (3.5–4.4)	3.68 (2.88–4.71)	<0.001	3.68 (2.82–4.81)	<0.001
Total	720/30,477	321,417	2.2 (2.1–2.4)				
HR per 1 SD increase				1.37 (1.31–1.43)	<0.001	1.34 (1.27–1.41)	<0.001
Korean Diabetic Cohort							
1st Quartile	13/1254	7661	1.7 (1.0–2.9)	1 (reference)		1 (reference)	
2nd Quartile	37/1253	7784	4.8 (3.4–6.6)	2.81 (1.49–5.29)	0.001	2.11 (1.10–4.04)	0.02
3rd Quartile	50/1254	7689	6.5 (4.9–8.6)	3.92 (1.27–3.31)	<0.001	2.54 (1.33–4.88)	0.005
4th Quartile	106/1253	6989	15.2 (12.5–18.3)	9.36 (5.26–16.67)	<0.001	5.56 (2.93–10.54)	<0.001
Total	206/5014	30,122	6.8 (6.0–7.8)				
HR per 1 SD increase				2.19 (1.89–2.53)	<0.001	1.94 (1.63–2.31)	<0.001

HR and 95% CI were estimated from Cox proportional hazard models.

*CI* confidence interval, *CKD* chronic kidney disease, *HR* hazard ratio, *eGFR* estimated glomerular filtration rate, *SD* standard deviation.

^a^Incidence rate per 1000 person-years.

^b^Adjusted model controlling for eGFR.

Subgroup analyses according to sex, age group, hypertension, diabetes, and eGFR levels are shown in Supplementary Fig. 1. In all subgroups, the Reti-CKD score was found to stratify future CKD risk with significant HRs.

### Reti-CKD score compared to the current standard of care

We compared the CKD prediction performance of Reti-CKD and eGFR-CKD scores in two longitudinal validation sets. (Table 3). In the Reti-CKD score model, compared to the eGFR-CKD score, C-statistic was significantly greater with a delta of 0.020 (95% CI, 0.011–0.029) in the UK Biobank and a delta of 0.024 (95% CI, 0.002–0.046) in the Korean Diabetic Cohort. The net reclassification index (NRI) revealed comparable findings (NRI in UK Biobank, 0.109 [95% CI, 0.44–0.156]; NRI in the Korean Diabetic cohort, 0.179 [95% CI, 0.017–0.292]).

**Table 3:** Prediction performance of Reti-CKD and eGFR-CKD scores.

	eGFR-CKD scores	Reti-CKD scores	*p* value
UK Biobank			
C-statistics	0.618 (0.598–0.638)	0.638 (0.618–0.658)	
Δ C-statistics	Ref	0.020 (0.011–0.029)	<0.001
NRI		0.109 (0.044–0.156)	<0.001
Diabetes cohort			
C-statistics	0.679 (0.642–0.717)	0.703 (0.664–0.742)	
Δ C-statistics	Ref	0.024 (0.002–0.046)	0.002
NRI		0.179 (0.017–0.292)	0.03

All 95% CIs and *p* values were determined using 1000 bootstrap samples with replacement.

*eGFR* estimated glomerular filtration rate, *eGFR-CKD scores* a conventional eGFR-based CKD risk score derived using the Cox proportional hazards model in the UK Biobank for comparison, *NRI* net reclassification index.

The following sensitivity analyses were in accordance with the main analysis: 1) Repeated analysis using the whole cohort population including participants with eGFR <90 mL/min/1.73 m^2^ or proteinuria at baseline (Supplementary Table 4) 2) Repeated analysis after dividing the UK Biobank cohort into groups with diabetes or hypertension and without (Supplementary Table 5). 3) Repeated analysis with only participants who were identified as Caucasian in the UK Biobank (Supplementary Table 6), 4) Landmark analysis (excluding participants with follow-up <1 year) (Supplementary Table 7), 5) Repeated analysis with CKD-EPI creatinine-cystatin equation determined eGFR, an alternative method for kidney function evaluation using serum creatinine levels (Supplementary Table 8).

## Discussion

In this study, we developed and validated the Reti-CKD score, a noninvasive chronic kidney disease risk stratification tool for people with preserved kidney function derived from retinal-based deep learning and clinical factors. Reti-CKD scores could stratify future CKD risk with a dose-dependent manner in two longitudinal studies, the UK Biobank and Korean Diabetic Cohort. In addition, when predicting CKD incidence, the C-statistic for the Reti-CKD score was significantly greater than the eGFR-based method in both cohorts.

First, we confirmed that the Reti-CKD score was superior to eGFR, the current standard for CKD screening, among people with preserved kidney function. Early detection is essential for preventing the progression to kidney failure requiring replacement therapy. Studies have shown that preventing proteinuria, an early sign of kidney disease, effectively lowers the risk of progressive kidney function decline^18^. In addition, those who were treated at an earlier disease phase were reported to develop advanced kidney disease at a slower rate^19–22^. However, among people with preserved kidney function, the efficacy of current biomarkers for detecting those at increased CKD risk is limited. The Reti-CKD score has potential as a unique, non-invasive risk score to help healthcare professionals identify and manage patients early. This can provide opportunities for active risk factor management, close monitoring, and timely treatment.

Second, the Reti-CKD score has several clinical benefits. Since the Reti-CKD score has superior performance in predicting CKD incidence compared to eGFR in people with normal kidney function, it can potentially be used as a simple and readily available screening tool in primary care or community clinics. The non-invasive nature of the Reti-CKD score makes it more suitable for population screening than conventional methods, allowing for accessible large-scale risk monitoring. In addition, Reti-CKD has also been shown to successfully predict the development of CKD in the entire population of the UK Biobank and Korean Diabetes Cohort which includes patients with underlying kidney disease. This suggests that Reti-CKD can be applied regardless of underlying kidney function. Moreover, clinical guidelines recommend routine retinal imaging to screen for retinopathy in patients with diabetes and hypertension, the two most common causes of CKD^23,24^. Therefore, accessibility will be further enhanced in these patient groups^25–27^.

Third, Reti-CKD effectively predicted CKD regardless of underlying hypertension or diabetes. Detecting kidney disease through retinal evaluation would be more feasible in patients who develop CKD due to systemic diseases. However, given that systemic conditions such as elevated blood pressure are also major risk factors for accelerating kidney function decline in patients with primary glomerulopathy, Reti-CKD may also operate appropriately in people developing CKD due to localized kidney diseases^28–30^. In addition, even in cases of localized kidney diseases, early-stage nephron loss may induce changes in the systemic milieu which could lead to retinal vascular pathology. Investigations showing that macrophage activation is promoted, even in early kidney disease stages, which induces systemic cholesterol accumulation through cholesterol efflux alteration support this possibility^31^.

Fourth, to the best of our knowledge, there are two prior studies related to the current topic. Sabanayagam et al. developed a retina-based deep-learning algorithm from a cross-sectional study that could diagnose CKD, but the prediction of future CKD events was not evaluated^17^. Further, Zhang et al. also proposed a deep-learning algorithm to predict CKD^32^. However, the total number of CKD cases was low in their validations, with a maximum of 6 years of follow-up (80 cases in the internal and 66 cases in the external test set). Moreover, risk stratification, in that study, was less apparent. Notably, in our exploratory analysis, the predictive power of the Reti-CKD score was >99% for both the UK Biobank and Korean Diabetic Cohort, a significant improvement over previous investigations^32^.

Interestingly, in the subgroup analyses, the impact of Reti-CKD predicting CKD was relatively greater among patients without diabetes than diabetic patients. Several explanations for this finding would be possible. First, more patients would be presenting CKD related retinal photograph findings among diabetic patients due to diabetic retinopathy, while retinal changes would be a comparably less common feature in non-diabetic patients^33^. This disproportion of retinal abnormality among groups may have resulted in a greater CKD prediction power for Reti-CKD in people without diabetes. Abnormal retinal changes in a group in which most people present normal retinal features may indicate a higher likelihood of concomitant kidney disease than those without retinal pathology. Second, in diabetes patients, other risk factors than retinal abnormalities may serve as powerful surrogates for kidney function status, such as blood glucose level or diabetes duration^34,35^. The presence of these other factors could have resulted in a relatively lower predictive capability of the Reti-CKD score. Nonetheless, although the predictive impact of Reti-CKD may slightly differ regarding the presence of diabetes, it should be noted that, in both patient populations, the effectiveness of Reti-CKD as a predictive marker was significant.

The major strength of this study is the development of a deep-learning algorithm using separate datasets from a Korean health screening center and validation of a new CKD risk score using two different cohorts (internal validation in the UK Biobank and external validation in the Korean Diabetic Cohort) with a sufficient number of CKD incidence. However, this study had several limitations. First, the primary outcome in the UK Biobank was defined according to claim codes from inpatient and general practice claim records. Due to human error and subjectivity, chances of CKD development being misdiagnosed in the claims data should be considered. Nonetheless, differences in CKD incidence across the four Reti-CKD score groups were significant lowering this possibility. This is because misdiagnosed cases would have been randomly distributed across the four risk groups. Additionally, sensitivity analyses also supported the association between the Reti-CKD score and CKD incidence, further reducing the likelihood that bias played a major role. Second, external validation had only been performed on the Korean Diabetic Cohort. There remains a need for further validating Reti-CKD scores in various disease populations and across different ethnicities.

In conclusion, we derived and validated a non-invasive CKD risk stratification tool, Reti-CKD score. For people with preserved kidney function, Reti-CKD score was more effective in the prediction of CKD incidence than conventional blood test-based method. Since access to retinal photography is increasing at community and primary care levels, Reti-CKD score has the potential to be adopted as a practical screening tool for primary CKD prevention.

## Methods

### Ethics statement

This study was conducted in accordance with the Declaration of Helsinki and approved by the institutional review board of Severance Hospital, Yonsei University Health System (4-2021-1174). Owing to the retrospective nature and use of de-identified data, the requirement for informed consent was waived for the Korean datasets. Written informed consent was obtained from the UK Biobank participants. UK Biobank resources were used under the application number 68428.

### Study population

**Fig. 3:**
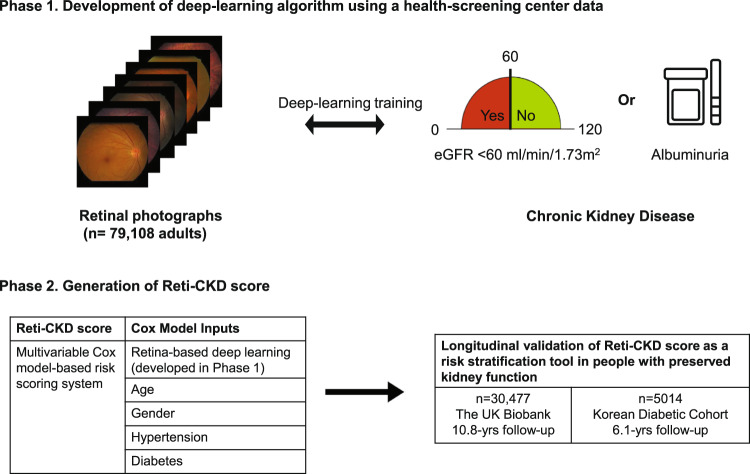
Study flow chart for derivation and validation of Reti-CKD score. In phase 1, health-screening center data was used for the development of deep-learning algorithm. In phase 2, data from longitudinal cohorts were utilized for derivation and validation of Reti-CKD score. Reti-CKD score was derived based on a Cox model using the UK Biobank cohort. The performance of Reti-CKD score was subsequently validated using the UK Biobank and Korean Diabetic Cohort.

In phase 1, a health screening center data were used for the development of deep-learning algorithm (Fig. 3). A development set including 158,216 retinal photographs was used to train the deep-learning algorithm. These retinal photographs were from 79,108 adults who had participated in health screening programs at a health screening center affiliated with Severance Hospital, South Korea.

In phase 2, data from longitudinal cohorts were used to derive and validate the Reti-CKD score. Reti-CKD score was derived using the UK Biobank cohort (*n* = 30,477). The performance of Reti-CKD score was subsequently validated using the UK Biobank and Korean Diabetic Cohort (*n* = 5014). The UK Biobank is a community-based prospective longitudinal study. The Korean Diabetic Cohort contains clinical data from a cohort of patients with type 2 diabetes who were treated at Severance Hospital or Gangnam Severance Hospital from April 2011 to August 2018.

Participants were eligible for this validation if clinical information for calculating eGFR and retinal photographs were available. Accordingly, participants with prevalent CKD, with eGFR <90 mL/min/1.73 m^2^, or with albuminuria (defined as urine albumin to creatinine ratio >30 mg/Cr in the UK Biobank and albumin ≥trace level on dip-stick urinalysis in the Korean Diabetic Cohort) were excluded. A detailed flowchart of the study population is shown in Supplementary Fig. 2.

### Retinal photography

In phase 1, retinal photographs were taken using three different retinal cameras in the development set: the AFP-210 nonmydriatic auto retinal camera (NIDEK Corporation, Aichi, Japan), TRC-NW8 nonmydriatic retinal camera (Topcon Corporation, Tokyo, Japan), and Nonmyd A-D (Kowa Co. Ltd., Shizuoka, Japan). In the development set, retinal photographs of all participants were taken, and blood tests were performed on the same day.

In phase 2, retinal photographs were taken at baseline using TRC-NW8 (Topcon Corporation, Tokyo, Japan) and KOWA VX-20 (Kowa, Chofu Factory, Japan) in the Korean Diabetic Cohort and Topcon 3D OCT-1000 Mark II (Topcon Corporation, Tokyo, Japan) in the UK Biobank.

### Development of deep-learning algorithm using retinal photographs: phase 1

In phase 1, contemplating that retinal microvascular signature associated with CKD could determine future risk of CKD, a deep-learning algorithm was trained (Fig. 3). The model inputs were retinal photographs. Ground truth was “absence versus presence” of CKD; CKD was defined as eGFR <60 mL/min/1.73 m^2^ or albuminuria and coded as a binary variable (no CKD versus CKD).

The utilized deep learning model was based on the ConvNeXT model. During the model training process, single images of each eye were separately inputted with a corresponding label. In the evaluation process, each image of the left and right eye had been assigned a probability score, and the average of these probability scores were considered as output of the examination, a process similar to ensemble learning with multiple models. This process showed improved performance than evaluating one image at a time each with a probability score. Our model design was almost identical to ConvNeXT, except for the dimension of the last fully connected layer, which was changed to one logit probability prediction. The logit that resulted from the last fully connected layer was converted to a probability with a sigmoid function, and we trained this model to minimize losses from the target and prediction. Further, we trained our model using the AdamW optimizer with a 0.0002 learning rate and cosine learning rate schedule for 50 epochs (Supplementary Fig. 3). For data augmentation, we used mixup, CutMix, RandAugment, contrast enhancement module, and random crop. Moreover, we not only adopted focal loss and exponential moving average but also used 384 × 384 size images. The cross-sectional performances of the deep learning algorithm’s prediction score in an internal validation set is provided in Supplementary Table 9. Once the deep-learning algorithm was trained, the probability of CKD presence could be calculated. This probability ranged from zero to one, with a high value indicating a high probability for having CKD. This “deep-learning-derived retina-CKD probability” was designed to calibrate the amount of association between the retinal microvascular signs and the presence of CKD. This deep-learning-derived retina-CKD probability alone without other clinical factors was tested to be capable of stratifying future CKD risk using two longitudinal cohorts of the UK Biobank and Korean Diabetic Cohort (Supplementary Table 10). Further, the deep-learning-derived retina-CKD probability was evaluated using Harrell’s c-statistic to show an incremental value over the eGFR model for CKD prediction (Supplementary Table 11).

### Development and validation of Reti-CKD score: phase 2

In phase 2, the predictability of the deep-learning-derived retina-CKD probability was further enhanced by integrating clinical factors (Fig. 3). The Reti-CKD score was derived using the Cox proportional hazards model in the UK Biobank. After fitting the Cox proportional hazards model to the UK biobank cohort, we obtained the coefficients of the covariates. The baseline survival probability in the UK biobank at 5 years was 0.9980896. Reti-CKD score was modeled to be a failure probability at 5 years. The Cox model included age, sex, hypertension, diabetes, and deep-learning-derived retina-CKD probability. The clinical factors were chosen to derive a parsimonious model because they can be obtained from questionnaires without additional invasive measures, such as blood tests (Supplementary Table 12).

For Reti-CKD risk score validation, four-tier CKD risk groups were proposed based on Reti-CKD score quartiles (1^st^, 2^nd^, 3^rd^, and 4^th^ quartile) in each cohort of the UK Biobank and Korean Diabetic Cohort. A conventional eGFR-based CKD risk score (i.e., eGFR-CKD score) was also derived using the Cox proportional hazards model in the UK Biobank for comparison (Supplementary Table 12). The performance of Reti-CKD and eGFR-CKD scores in prediction of CKD events were assessed in the UK Biobank and Korean Diabetic Cohort, respectively.

### CKD incidence definition

In the UK Biobank, CKD incidence was defined according to the tenth revision of the International Statistical Classification of Diseases and Related Health Problems (ICD-10) codes and the Office of Population Censuses and Surveys Classification of Interventions and Procedures version 4 (OPCS-4) codes in primary care settings, hospital inpatient data, and death register records (Supplementary Table 13) The follow-up period began at the date of the first assessment and ended with death, CKD diagnosis, or the end of follow-up, whichever occurred first.

In the Korean Diabetic Cohort, CKD incidence was defined as two consecutive eGFR values of <60 mL/min/1.73 m^2^, the first of which was used as the index date. The follow-up period for each patient began when their retinal photographs were taken and ended on the date of CKD diagnosis or the last creatinine measurement.

### Definition of covariates

The eGFR was calculated from serum creatinine using the Chronic Kidney Disease Epidemiology Collaboration equation (CKD-EPI)^36^. In the UK Biobank, diabetic history was determined by ICD-10 codes in any primary care setting and hospital inpatient data. Hypertension was defined as the use of antihypertensive medications in any primary care setting, hospital inpatient data, and self-reported medical history records. Similarly, in the Korean Diabetic Cohort, hypertension was established for patients on antihypertensive medication according to outpatient and inpatient prescription data.

### Saliency maps

To explain how the deep learning model works, saliency maps were generated. We used guided backpropagation, which uses gradients of class probability for each image pixel, to demonstrate how pixels can affect the prediction results of the model^37^. Further, to obtain a more robust and clear visualization, we used the SmoothGrad technique, which averages gradients from images with random noise^38^.

### Statistical analysis

Python 3.7 was used for development of the deep-learning algorithm, and Stata version 16.1 (Stata Corp, TX, USA) and R version 5.0.3 (R Foundation, Vienna, Austria) were used for survival analysis and model performance assessment. Statistical significance was set at *P* < 0.05. Descriptive statistics were provided for all datasets including health screening data, the UK Biobank, and the Korean Diabetic Cohort.

In the UK Biobank, each participant was followed up to 11.6 years from the date of the initial visit to the last follow-up date (February 28, 2021) or the date of CKD diagnosis. In the Korean Diabetic Cohort, each patient was followed up to 14.0 years from the date of the initial visit to the last follow-up date (February 28, 2022) or the date of CKD diagnosis. The cumulative incidence of CKD was evaluated across the quartiles defined by the Reti-CKD score using the Kaplan–Meier method and Cox proportional hazards model to estimate HRs. The eGFR-adjusted model included the baseline eGFR as a covariate.

The prognostic value of the Reti-CKD and eGFR-CKD scores in predicting CKD incidence was assessed using Harrell’s C-statistic and NRI^39,40^. Further, to obtain 95% CIs, we used a non-parametric bootstrap procedure with 1000 samples.

For sensitivity analyses, we additionally evaluated the performance of Reti-CKD in the entire population including prevalent CKD. Second, we repeated our survival analysis with participants with and without underlying diabetes or hypertension to evaluate predictability among different CKD etiologies. Third, analysis was done with participants identified as Caucasians in the UK Biobank. Fourth, landmark analysis was conducted using both cohorts after excluding subjects with a follow-up period of <1 year. Finally, analysis was done with eGFR converted form the CKD-EPI creatinine-cystatin equation^41^.

## Supplementary information

Supplementary Material

## Data Availability

The UK Biobank test dataset was obtained from the UK Biobank (application number 68428). The raw UK Biobank data-including the retinal photograph data reported here-are made available to researchers from universities and other research institutions with genuine research inquiries following IRB and UK Biobank approval. Data from South Korea are available to researchers who meet the criteria for access to confidential data. Data can be accessed upon request from the corresponding author, Jung Tak Park, Department of Internal Medicine, College of Medicine, Yonsei University, Seoul, South Korea (JTPARK@yuhs.ac).

## Code availability

Code cannot be shared publicly because Reti-CKD is a commercial product. However, Reti-CKD are available to researchers who meet the criteria for access to confidential data; requests should be made to Mediwhale Inc. (info@mediwhale.com).
