# Supplementary material for: Non-invasive chronic kidney disease risk stratification tool derived from retina-based deep learning and clinical factors
Source: NPJ Digit Med. 2023 Jun 17;6:114. doi: 10.1038/s41746-023-00860-5 (PMC10276847; doi:10.1038/s41746-023-00860-5)
Supplement: Supplementary file 1 — Supplementary Material [file 41746_2023_860_MOESM1_ESM.docx]

# Supplementary Document

Non-invasive Chronic Kidney Disease Risk Stratification Tool Derived from Retina-based Deep Learning and Clinical Factors

Supplement to: Joo Y et al.

**Supplementary Figure 1.** Subgroup Analysis of the Reti-CKD Score for Incident Chronic Kidney Disease

**Supplementary Figure 2.** Derivation of Study Cohort.

**Supplementary Figure 3.** Learning curve for [Convolutional Neural Network](https://www.tensorflow.org/tutorials/images/cnn) model

**Supplementary Table 1.** Chronic Kidney Disease Prevalence in the Health Screening data

**Supplementary Table 2.** Baseline Characteristics of the UK Biobank According to Reti-CKD Scores

**Supplementary Table 3.** Baseline Characteristics of the Korean Diabetic Cohort According to Reti-CKD Scores

**Supplementary Table 4.** Sensitivity Analysis- Whole Screening Population Results

Supplementary Table 5. Sensitivity Analysis- Predictive performance of Reti-CKD according to Diabetes or Hypertension at enrollment in UK Biobank

**Supplementary Table 6.** Sensitivity Analysis- Analysis Limited to Caucasian Ethnicity Participants in UK Biobank

**Supplementary Table 7.** Sensitivity Analysis- Landmark Analysis Including Patients Who Were Followed for More Than 1 Year Post-recruitment

**Supplementary Table 8.** Sensitivity Analysis- Creatinine-cystatin Based Estimated Glomerular Filtration Ratio in Available Participants in UK Biobank

**Supplementary Table 9.** Prediction Performance of Deep-learning-derived retina-CKD probability for CKD Presence

**Supplementary Table 10.** Association between Chronic Kidney Disease Development and Deep-learning-derived retina-CKD probability

**Supplementary Table 11.** Prediction Performance of retina-based deep-learning model and eGFR model

Supplementary Table 12. Formulae for Prediction Models

Supplementary Table 13. Codes Used for Operational Chronic Kidney Disease Definition in the UK Biobank

Supplementary Figure 1. Subgroup Analysis of the Reti-CKD Score for Incident Chronic Kidney Disease. Subgroup analyses were performed according to sex, age, hypertension, diabetes, and eGFR using multivariable Cox proportional hazard model. Hazard ratio (HR) trends were provided with 95% confidence intervals (CI). HR trends was used to test for association for each outcome across increasing quartiles for Reti-CKD. *HTN* hypertension, *eGFR* estimated glomerular filtration rate, *HR* hazard ratio, *CI* confidence interval.


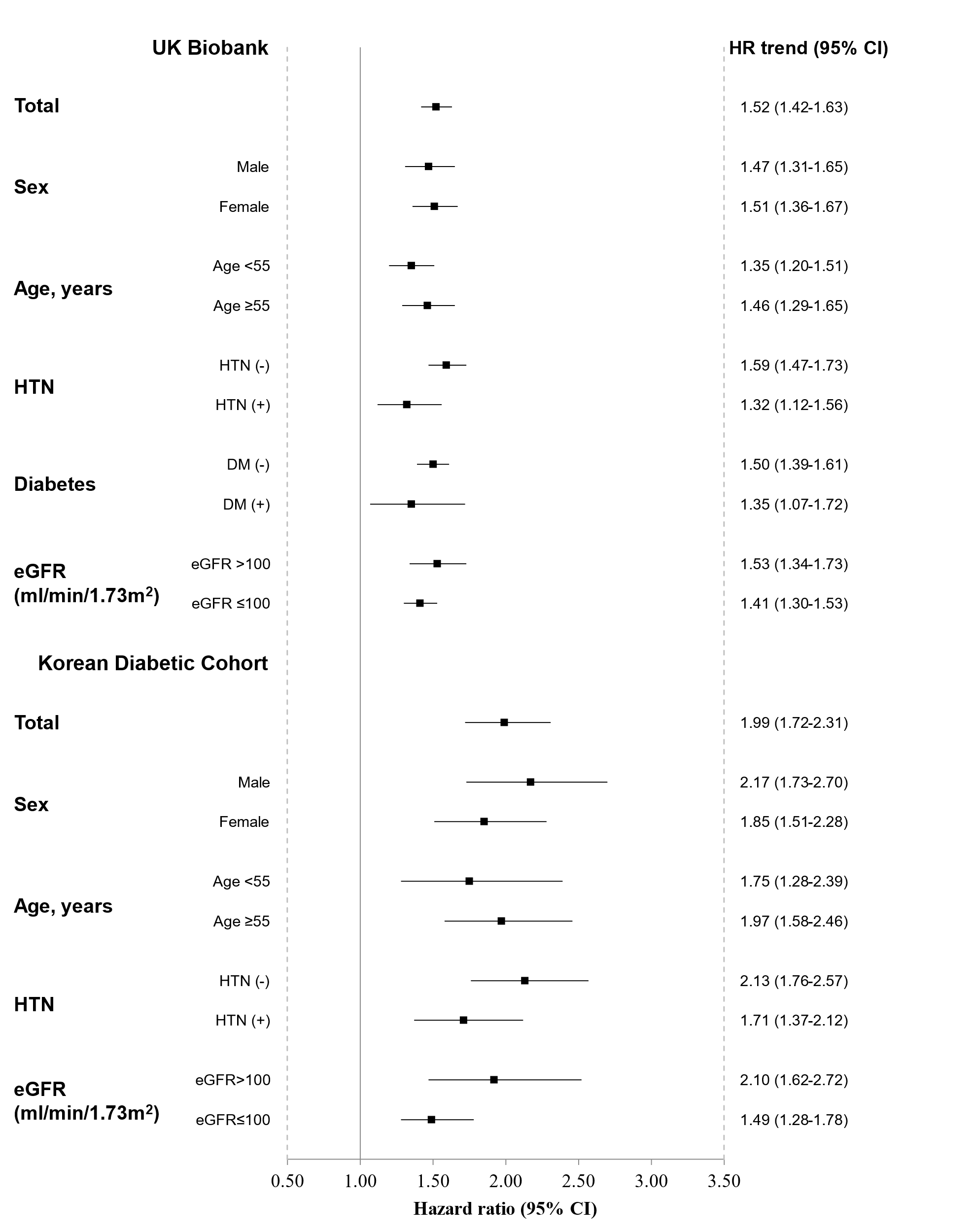


Supplementary Figure 2. Derivation of Study Cohort. *CKD* chronic kidney disease, *eGFR* estimated glomerular filtration rate.


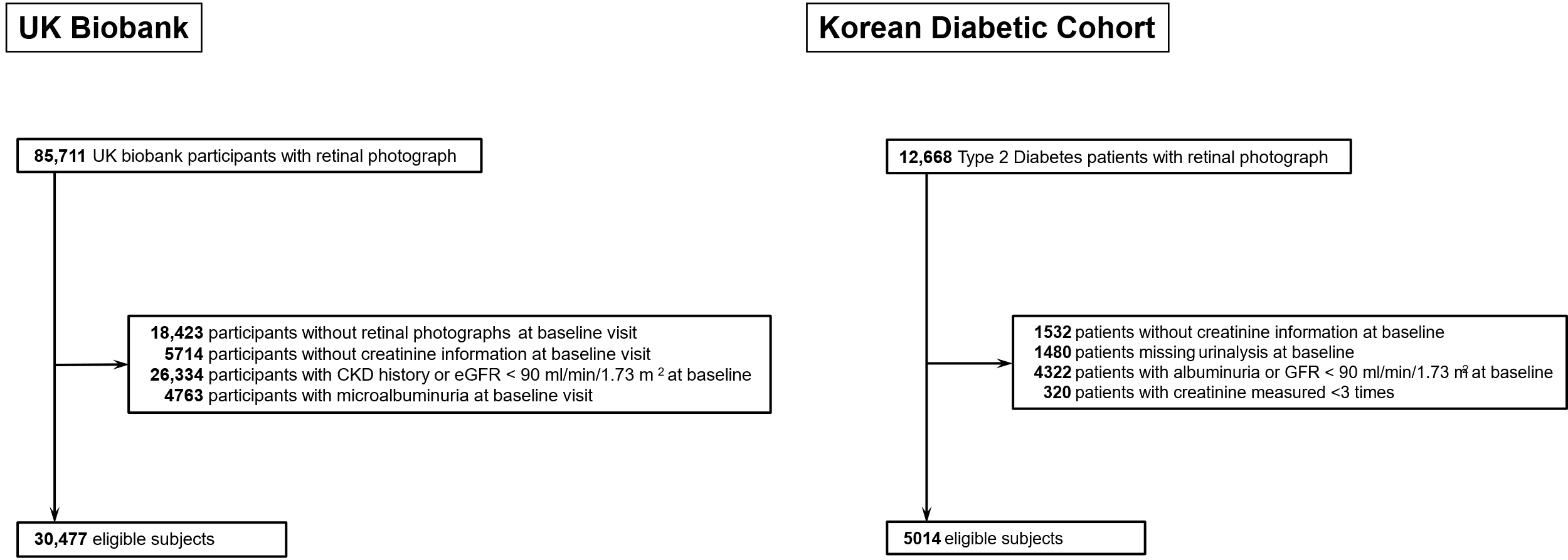


**Supplementary Figure 3. Learning curve for** [**Convolutional Neural Network**](https://www.tensorflow.org/tutorials/images/cnn) **model. a** Learning curve of the training of the neural network with focal loss and **b** area under the curve.

*AUROC* area under the receiver operating characteristic.


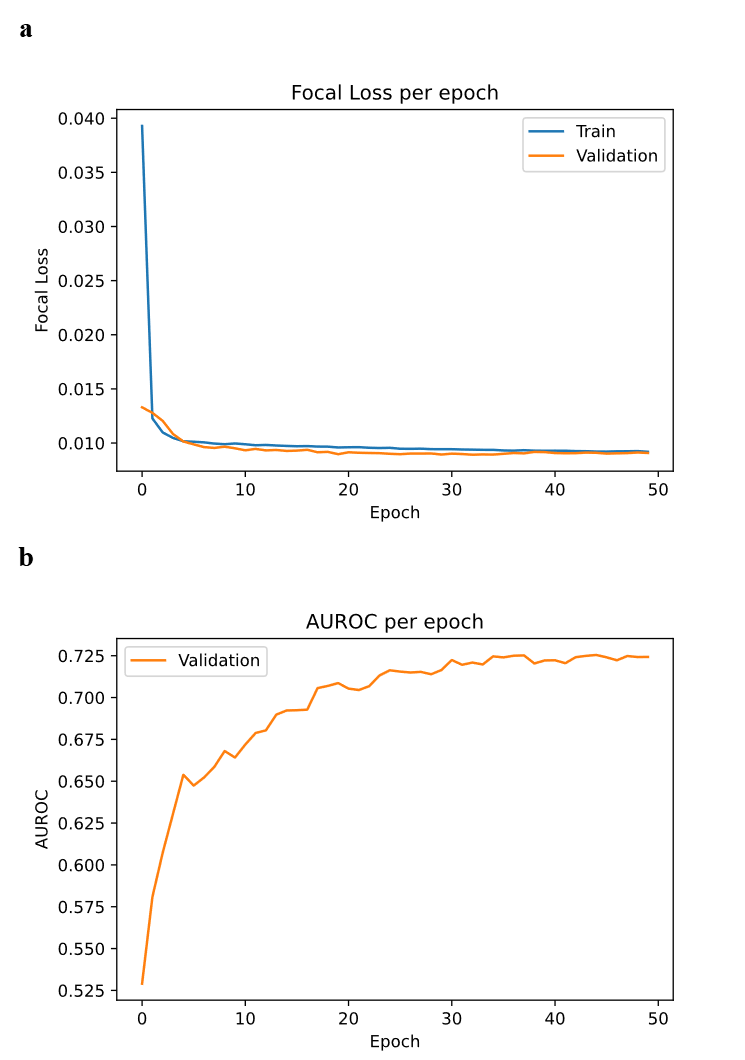


Supplementary Table 1. Chronic Kidney Disease Prevalence in the Health screening data

|  | Health screening data  (n=79,108) |
| --- | --- |
| CKD, No. (%) | 9934 (12.6) |
| eGFR < 60 mL/min/1.73 m^2^ (%) | 680 (0.86) |
| Proteinuria (%) | 9485 (12.0) |
| Data are expressed as proportion n (%).  *CKD* chronic kidney disease, *eGFR* estimated glomerular filtration rate. | |

Supplementary Table 2. Baseline Characteristics of the UK Biobank According to Reti-CKD Scores

|  | Overall  (n=30,477) | Reti-CKD scores | | | |
| --- | --- | --- | --- | --- | --- |
|  |  | 1^st^ Quartile  (n=7620) | 2^nd^ Quartile  (n=7619) | 3^rd^ Quartile  (n=7619) | 4^th^ Quartile  (n=7619) |
| Age, mean (SD), years | 54.2 ± 8.1 | 45.6 ± 4.0 | 51.4 ± 5.7 | 57.6 ± 5.8 | 62.1 ± 4.6 |
| Male, No. (%) | 13,617 (44.7) | 1293 (17.0) | 3274 (43.0) | 3611 (47.4) | 5439 (71.4) |
| Diabetes, No. (%) | 1293 (4.2) | 7 (0.1) | 61 (0.8) | 204 (2.7) | 1021 (13.4) |
| Hypertension, No. (%) | 4050 (13.3) | 504 (6.6) | 734 (9.6) | 1156 (15.2) | 1656 (21.7) |
| eGFR, mean (SD), mL/min/1.73 m^2^ | 99.4 ± 6.6 | 103.8 ± 7.2 | 100.5 ± 6.2 | 97.6 ± 5.3 | 95.6 ± 4.5 |
| Data are expressed as mean ± standard deviation or proportion n (%).  *SD* standard deviation*, eGFR* estimated glomerular filtration rate. | | | | | |

Supplementary Table 3. Baseline Characteristics of the Korean Diabetic Cohort According to Reti-CKD Scores

|  | Overall  (n=5,014) | Reti-CKD scores | | | |
| --- | --- | --- | --- | --- | --- |
|  |  | 1^st^ Quartile  (n=1254) | 2^nd^ Quartile  (n=1253) | 3^rd^ Quartile  (n=1254) | 4^th^ Quartile  (n=1253) |
| Age, mean (SD), years | 55.1 ± 10.5 | 42.9 ± 8.4 | 53.5 ± 5.9 | 59.3 ± 5.4 | 64.8 ± 6.4 |
| Male, No. (%) | 2814 (56.1) | 540 (43.1) | 658 (52.5) | 772 (61.6) | 844 (67.4) |
| Hypertension, No. (%) | 1400 (27.9) | 296 (23.6) | 338 (27.0) | 351 (28.0) | 415 (33.1) |
| eGFR, mean (SD), mL/min/1.73 m^2^ | 102.5 ± 9.1 | 110.9 ± 10.2 | 102.8 ± 6.6 | 99.2 ± 6.0 | 97.0 ± 5.8 |
| Data are expressed as mean ± standard deviation or proportion n (%).  *SD* standard deviation*, eGFR* estimated glomerular filtration rate. | | | | | |

Supplementary Table 4. Sensitivity Analysis- Whole Screening Population Results

|  | eGFR-CKD scores | Reti-CKD scores | *p* value |
| --- | --- | --- | --- |
| UK Biobank |  |  |  |
| C-statistics | 0.619 (0.603–0.634) | 0.627 (0.612–0.643) |  |
| Δ C-statistics | Ref | 0.008 (0.001–0.016) | 0.003 |
| Diabetes cohort |  |  |  |
| C-statistics | 0.685 (0.670–0.700) | 0.742 (0.729–0.756) |  |
| Δ C-statistics | Ref | 0.057 (0.048–0.067) | <0.001 |
| All 95% CIs and *p* values were determined using 1000 bootstrap samples with replacement.  *eGFR* estimated glomerular filtration rate, *eGFR-CKD scores* conventional eGFR-based CKD risk score derived using the Cox proportional hazards model in the UK Biobank for comparison. | | | |

Supplementary Table 5. Sensitivity Analysis- Predictive performance of Reti-CKD according to Diabetes or Hypertension at enrollment in UK Biobank

|  | eGFR-CKD scores | Reti-CKD scores | *p* value |
| --- | --- | --- | --- |
| Participants with DM and/or HTN |  |  |  |
| C-statistics | 0.549 (0.526–0.572) | 0.568 (0.545–0.591) |  |
| Δ C-statistics |  | 0.019 (0.008–0.030) | 0.001 |
| Participants without any DM or HTN |  |  |  |
| C-statistics | 0.564 (0.510–0.617) | 0.589 (0.534–0.617) |  |
| Δ C-statistics |  | 0.025 (0.002–0.048) | 0.001 |
| Participants with diabetes at baseline and diagnosed during follow-up period were allocated to diabetic patients in current analysis. All 95% CIs and *p* values were determined using 1000 bootstrap samples with replacement.  *DM* diabetes, *HTN* hypertension, *eGFR* estimated glomerular filtration rate, *eGFR-CKD scores* conventional eGFR-based CKD risk score derived using the Cox proportional hazards model in the UK Biobank for comparison. | | | |

Supplementary Table 6. Sensitivity Analysis- Analysis Limited to Caucasian Ethnicity Participants in UK Biobank

| Reti-CKD score | CKD events/N | Person-years | Incidence rate^a^ | Crude model  HR (95% CI) | *p* value | eGFR-adjusted model^b^  HR (95% CI) | *p* value |
| --- | --- | --- | --- | --- | --- | --- | --- |
| 1st Quartile | 80/6831 | 73,315 | 1.1 (0.9–1.4) | 1 (reference) |  | 1 (reference) |  |
| 2nd Quartile | 105/6831 | 72,702 | 1.4 (1.2–1.7) | 1.31 (0.98–1.75) | 0.07 | 1.30 (0.97–1.74) | 0.08 |
| 3rd Quartile | 195/6831 | 71,661 | 2.7 (2.4–3.1) | 2.46 (1.90–3.19) | <0.001 | 2.42 (1.84–3.18) | <0.001 |
| 4th Quartile | 283/6831 | 70,246 | 4.0 (3.6–4.5) | 3.60 (2.81–4.62) | <0.001 | 3.52 (2.68–4.64) | <0.001 |
| Total | 663/27,324 | 287,924 | 2.3 (2.1–2.5) |  |  |  |  |
| HR per 1 SD increase |  |  |  | 1.37 (1.31–1.44) | <0.001 | 1.34 (1.27–1.42) | <0.001 |
| ^a^Incidence rate per 1,000 person-years. ^b^Adjusted model controlling for eGFR. *HR* hazard ratio, *CI* confidence interval, *eGFR* creatinine-based estimated glomerular filtration ratio, *SD* standard deviation. | | | | | | | |

Supplementary Table 7. Sensitivity Analysis- Landmark Analysis Including Patients Who Were Followed for More than 1 Year Post-recruitment

| Reti-CKD score | CKD events/N | Person-years | Incidence rate^a^ | Crude model  HR (95% CI) | *p* value | eGFR-adjusted model^b^  HR (95% CI) | *p* value |
| --- | --- | --- | --- | --- | --- | --- | --- |
| UK Biobank |  |  |  |  |  |  |  |
| 1st Quartile | 69/7576 | 81,521 | 0.8 (0.7–1.1) | 1 (reference) |  | 1 (reference) |  |
| 2nd Quartile | 98/7576 | 80,912 | 1.2 (1.0–1.5) | 1.41 (1.03–1.92) | 0.03 | 1.38 (1.01–1.88) | 0.04 |
| 3rd Quartile | 172/7576 | 80,045 | 2.1 (1.9–2.5) | 2.49 (1.88–3.30) | <0.001 | 2.39 (1.78–3.21) | <0.001 |
| 4th Quartile | 245/7576 | 78,861 | 3.1 (2.7–3.5) | 3.56 (2.73–4.66) | <0.001 | 3.38 (2.52–4.54) | <0.001 |
| Total | 584/30,303 | 321,340 | 1.8 (1.7–2.0) |  |  |  |  |
| HR per 1 SD increase |  |  |  | 1.36 (1.29–1.43) | <0.001 | 1.32 (1.25–1.40) | <0.001 |
| Korean Diabetic Cohort |  |  |  |  |  |  |  |
| 1st Quartile | 11/1182 | 7666 | 1.4 (0.8–2.6) | 1 (reference) |  | 1 (reference) |  |
| 2nd Quartile | 34/1181 | 7693 | 4.4 (3.2–6.2) | 3.05 (1.55–6.02) | 0.001 | 2.33 (1.16–4.68) | 0.02 |
| 3rd Quartile | 45/1181 | 7607 | 5.9 (4.4–7.9) | 4.18 (2.16–8.08) | <0.001 | 2.78 (1.38–5.61) | 0.004 |
| 4th Quartile | 97/1181 | 6996 | 13.9 (11.4–16.9) | 10.27 (5.50–19.17) | <0.001 | 6.29 (3.15–12.53) | <0.001 |
| Total | 187/4725 | 29,963 | 6.2 (5.4–7.2) |  |  |  |  |
| HR per 1 SD increase |  |  |  | 2.19 (1.89–2.55) | <0.001 | 1.96 (1.63–2.35) | <0.001 |
| ^a^Incidence rate per 1000 person-years. ^b^Adjusted model controlling for eGFR. HR and 95% CI were estimated from Cox proportional hazard models. *HR* hazard ratio, *CI* confidence interval, *eGFR* creatinine-based estimated glomerular filtration ratio, *SD* standard deviation. | | | | | | | |

Supplementary Table 8. Sensitivity Analysis- Creatinine-cystatin Based Estimated Glomerular Filtration Ratio in Available Participants in UK Biobank

| Reti-CKD score | CKD events/N | Person-years | Incidence rate^a^ | Crude model  HR (95% CI) | *p* value | eGFR-adjusted model^b^  HR (95% CI) | *p* value |
| --- | --- | --- | --- | --- | --- | --- | --- |
| 1st Quartile | 62/6430 | 69,126 | 0.9 (0.7–1.2) | 1 (reference) |  | 1 (reference) |  |
| 2nd Quartile | 115/6429 | 68,401 | 1.7 (1.4–2.0) | 1.85 (1.36–2.53) | <0.001 | 1.79 (1.31–2.44) | <0.001 |
| 3rd Quartile | 159/6430 | 67,781 | 2.3 (2.0–2.7) | 2.56 (1.91–3.44) | <0.001 | 2.31 (1.78–3.25) | <0.001 |
| 4th Quartile | 245/6429 | 66,724 | 3.7 (3.2–4.2) | 3.96 (2.99–5.23) | <0.001 | 3.63 (2.71–4.87) | <0.001 |
| Total | 581/25,718 | 272,032 | 2.1 (2.0–2.3) |  |  |  |  |
| HR per 1 SD increase |  |  |  | 1.34 (1.27–1.40) | <0.001 | 1.33 (1.27–1.40) | <0.001 |
| Participants with both creatinine and cystatin C levels were included in this analysis. HR and 95% CI were estimated from Cox proportional hazard models. ^a^Incidence rate per 1000 person-years. ^b^Risk-adjusted controlling for cystatin-creatinine-based eGFR.  *HR* hazard ratio, *CI* confidence interval, *eGFR* cystatin-creatinine-based estimated glomerular filtration ratio, *SD* standard deviation. | | | | | | | |

Supplementary Table 9. Prediction Performance of Deep-learning-derived retina-CKD probability for CKD Presence

| Metrics | Algorithm derived from all retinal photograph | Algorithm derived from retinal photograph with DR | Algorithm derived from retinal photograph without DR |
| --- | --- | --- | --- |
| AUC | 0.727 (0.695–0.756) | 0.708 (0.575–0.641) | 0.719 (0.690–0.748) |
| Accuracy | 0.779 (0.617–0.836) | 0.660 (0.535–0.785) | 0.762 (0.651–0.873) |
| Sensitivity | 0.589 (0.487–0.732) | 0.561 (0.451–0.671) | 0.602 (0.417–0.787) |
| Specificity | 0.783 (0.616–0.842) | 0.634 (0.474–0.794) | 0.766 (0.650–0.882) |
| Deep learning algorithms were derived from all retinal photograph, retinal photograph with DR, and retinal photograph without DR. The algorithm derived from all retinal photograph was selected for deep-learning-derived retina-CKD probability.  *DR* diabetic retinopathy, *AUC* area under the receiver operating characteristic curve, *CI* confidence interval. | | | |

Supplementary Table 10. Association between Chronic Kidney Disease Development and Deep-learning-derived retina-CKD probability

| Deep-learning-derived retina-CKD probability | CKD events/n | Incidence | Age-adjusted and sex-adjusted model  HR (95% CI) | Risk-adjusted model^a^  HR (95% CI) |
| --- | --- | --- | --- | --- |
| UK Biobank |  |  |  |  |
| Total | 720/30,477 | 2.4 (2.1–2.4) | 1.06 (1.04–1.08) | 1.05 (1.04–1.07) |
| Korean Diabetic Cohort |  |  |  |  |
| Total | 206/5014 | 6.8 (6.0–7.8) | 1.05 (1.03–1.06) | 1.05 (1.03–1.06) |
| ^a^Risk-adjusted model controlling for age, sex, hypertension, and diabetes. HR and 95% CI were estimated from Cox proportional hazard models. *CKD* chronic kidney disease, *HR* hazard ratio, *CI* confidence interval. | | | | |

Supplementary Table 11. Prediction Performance of retina-based deep-learning model and eGFR model

|  | eGFR model | Deep-learning model | *p* value | eGFR + deep-learning model | *p* value |
| --- | --- | --- | --- | --- | --- |
| UK Biobank |  |  |  |  |  |
| C statistics | 0.612 (0.591–0.632) | 0.633 (0.613–0.654) |  | 0.634 (0.623–0.654) |  |
| Δ C-statistics | Ref | 0.021 (0.011–0.032) | <0.001 | 0.021 (0.011–0.032) | <0.001 |
| Korean Diabetes cohort |  |  |  |  |  |
| C statistics | 0.702 (0.666–0.740) | 0.715 (0.677–0.752) |  | 0.718 (0.680–0.756) |  |
| Δ C-statistics | Ref | 0.013 (-0.004–0.029) | 0.15 | 0.016 (0.002–0.029) | 0.02 |
| All models were adjusted for age, sex, hypertension, and diabetes. Numbers indicate Harrell’s C statistics followed by 95% confidence intervals in parentheses.  *eGFR* estimated glomerular filtration rate. | | | | | |

Supplementary Table 12. Formulae for Prediction models

| Prediction model | Formula for predicted risk of the primary renal outcome |
| --- | --- |
| Reti-CKD score  (The Cox model included age, sex, hypertension, diabetes, and deep-learning-derived retina-CKD probability.) | $Predicted risk (time 5 year) =1-{0.9980896}^{Exp[LP]}$  LP=0.0546351*Retinal photograph-based prediction score *100 +0·0357466*Age-0.3317242*Female-0.1207903*Hypertension +0.4679712*Diabetes |
| eGFR-CKD score  (The Cox model included age, sex, hypertension, diabetes, and eGFR.) | $Predicted risk (time 5 year) =1-{0.9980896}^{Exp[LP]}$  LP=-0·0031299*eGFR +0.0438325*Age -0.3750974*Female-0.0823699*Hypertension +0.5181706*Diabetes |
| *LP* linear predictor, *eGFR* estimated glomerular filtration rate. | |

Supplementary Table 13. Codes Used for Operational Chronic Kidney Disease Definition in the UK Biobank

| Prediction model | Codes |
| --- | --- |
| International classification of diseases (ICD-10) codes: | I12.0, I12.9, I13.0, I13.1, I13.2, I13.9, E11.2, E10.2, E12.2, E13.2, E14.2, N18, T86.1, and Z94.0 |
| OPCS-4 codes | L74.1, L74.2, L74.3, L74.4, L74.5, L74.6, L74.8, L74.9, M01.2, M01.3, M01.4, M01.5, M01.8, M01.9, M02.3, M08.4, M17.2, M17.4, M17.8, M17.9, X40.2, X40.5, X40.6, X41.1, and X41.2 |
